# Wearable Technologies and AI at the Far Edge for Chronic Heart Failure Prevention and Management: A Systematic Review and Prospects

**DOI:** 10.3390/s23156896

**Published:** 2023-08-03

**Authors:** Angela-Tafadzwa Shumba, Teodoro Montanaro, Ilaria Sergi, Alessia Bramanti, Michele Ciccarelli, Antonella Rispoli, Albino Carrizzo, Massimo De Vittorio, Luigi Patrono

**Affiliations:** 1 Department of Engineering for Innovation, University of Salento, 73100 Lecce, Italy; angela.shumba@unisalento.it (A.-T.S.); teodoro.montanaro@unisalento.it (T.M.); ilaria.sergi@unisalento.it (I.S.); massimo.devittorio@unisalento.it (M.D.V.); 2 Istituto Italiano di Tecnologia, Centre for Biomolecular Nanotechnologies, 73010 Arnesano, Italy; 3 Dipartimento di Medicina, Chirurgia e Odontoiatria “Scuola Medica Salernitana” (DIPMED), University of Salerno, 84081 Baronissi, Italy; abramanti@unisa.it (A.B.); mciccarelli@unisa.it (M.C.); antonellarispoli@gmail.com (A.R.); acarrizzo@unisa.it (A.C.)

**Keywords:** wearables, chronic heart failure, edge AI, on-device AI systematic literature review, personalized health, health digital twin, IoT, smart health infrastructures, cardiovascular diseases, internet of wearable things, internet of medical things

## Abstract

Smart wearable devices enable personalized at-home healthcare by unobtrusively collecting patient health data and facilitating the development of intelligent platforms to support patient care and management. The accurate analysis of data obtained from wearable devices is crucial for interpreting and contextualizing health data and facilitating the reliable diagnosis and management of critical and chronic diseases. The combination of edge computing and artificial intelligence has provided real-time, time-critical, and privacy-preserving data analysis solutions. However, based on the envisioned service, evaluating the additive value of edge intelligence to the overall architecture is essential before implementation. This article aims to comprehensively analyze the current state of the art on smart health infrastructures implementing wearable and AI technologies at the far edge to support patients with chronic heart failure (CHF). In particular, we highlight the contribution of edge intelligence in supporting the integration of wearable devices into IoT-aware technology infrastructures that provide services for patient diagnosis and management. We also offer an in-depth analysis of open challenges and provide potential solutions to facilitate the integration of wearable devices with edge AI solutions to provide innovative technological infrastructures and interactive services for patients and doctors.

## 1. Introduction

Heart failure affects approximately 26 million people worldwide and causes over 1 million hospitalizations in Europe and North America alone, causing a significant burden to healthcare systems [1,2,3,4]. Several cardiac events, including arrhythmias (abnormality in heart rhythms) and myocardial infarctions (MIs), aka (heart attacks), eventually lead to chronic heart failure (CHF). Therefore, the prevention and monitoring of these events are crucial, particularly in high-risk groups like diabetics, people with hypertension, the elderly, and obese individuals [1,5]. Using unobtrusive, continuous monitoring technologies can help medical professionals devise solutions to slow disease progression and prolong the lives of diagnosed CHF patients. For instance, detecting and treating Atrial Fibrillation (AFib), a common occurrence in CHF patients, can stabilize ventricular function and reduce mortality [1]. For this reason, much research is being directed towards developing innovative continuous monitoring technology solutions to support the diagnosis and treatment of this class of diseases and other chronic illnesses that affect a significant portion of the world’s population [3,6]. Various innovative technologies have been applied to the healthcare sector, promoting the development of trustworthy solutions that can provide reliable personalized care to augment the burdened healthcare systems [7,8]. In recent solutions, the artificial intelligence (AI) paradigm has been used to perform the accurate and intuitive analysis of health data, predict health status, and generate warnings to facilitate proactive and timely interventions [3,5]. Legacy AI-based solutions leverage primarily cloud-based architectures, in which data collected by wearable sensors must be transferred to a remote server for elaboration and analysis [9]. Such cloud-based solutions require continuous wireless data transfer using vulnerable communication channels [6], which is one of the primary reasons why patients and doctors are reluctant to accept these technologies. The resulting reluctance is usually due to privacy, ease of use, perceived risk, and efficacy issues [10,11,12,13]. Therefore, the edge computing and near-sensor processing paradigms have been introduced to solve some of these issues by performing varying levels of computation, data processing, and analysis tasks closer to the data sources. In some cases, the tasks are performed directly on the devices worn by the patients, or on nearby small devices [3,5]. Additionally, the combination of AI and edge computing has introduced a new concept called edge intelligence [14], which enables intuitive and dynamic data processing and pattern recognition very close to the data source. Adopting wearable-based edge intelligence for the prevention and management infrastructures within the chronic heart failure context can promote and guarantee efficient at-home healthcare services in emergencies [15]. To this aim, various works presented in the literature have investigated the main advantages of deploying edge intelligence techniques on wearable devices. However, a thorough literature search revealed the absence of a comprehensive analysis from a clinical and technology perspective, highlighting solutions applied to the CHF context and outlining their effect on patients, medical professionals, and other concerned parties.

This paper, therefore, provides an up-to-date comprehensive collation and analysis of existing edge AI solutions deployed in wearables for CHF-prevention or management-applications. With this literature analysis, we delineate the extent to which edge AI is implemented to improve the efficacy of technology applied to the CHF context. We also identify research gaps and provide recommendations to potentially improve the efficiency of the edge AI paradigm in wearable-based CHF prevention and management.

The rest of the paper is organized as follows: Section 2 provides background information related to CHF and the technologies applied to the domain to provide the reader with the necessary background information from a medical and technological perspective; Section 3 presents a summary of literature surveys highlighting the application of technology solutions to CHF; Section 4 presents the applied research methodology and highlights the main contributions of this work; Section 5 discusses the results of the literature survey; Section 6 presents the main observations and offers potential solutions to the identified research gaps and future research directions; and, finally, conclusions and recommendations for future developments are presented in Section 7.

## 2. Background

CHF is increasingly becoming a social, health, and economic burden because of population growth, ageing, and the augmented prevalence of comorbidities. A technological approach to this disease may be essential to reduce this burden. Understanding the mechanics of CHF is crucial for developing relevant and effective technology intervention solutions to improve the primary and secondary prevention of cardiovascular events and heart failure re-actualization. Therefore, this section provides some medical facts about CHF, including how it is diagnosed and related to other cardiovascular diseases (CVDs).

### 2.1. Chronic Heart Failure

#### 2.1.1. What Is It?

CHF is a complex clinical syndrome resulting from the structural modifications and/or functional limitations of the heart that determine a reduction in the cardiac output or augmentation of the pressure within the atrial and ventricular chambers at rest or during physical activities. The typical clinical signs and symptoms of heart failure are peripheral oedema, pulmonary crackles, dyspnea, and fatigue [16]. In addition, the New York Heart Association (NYHA) criteria classify CHF according to the severity of the symptoms and the physical activity threshold at which dyspnea and fatigue appear. The NYHA class is commonly used in clinical practice because it is an independent predictor of mortality and rehospitalization [17]. Etiologically, the common cause of CHF is myocardial infarction or chronic coronary syndrome. Non-ischemic causes of CHF are distinguished in valvular heart disease, arterial hypertension, infiltrative cardiac disease, myocarditis, and familiar and genetic cardiomyopathy [18].

#### 2.1.2. How Is It Diagnosed?

The suspicion of CHF starts from evaluating clinical signs and symptoms of heart failure, which may vary according to the patient’s medical history of arterial hypertension, chronic coronary syndrome, diabetes mellitus, dyslipidemia, chronic kidney disease, and alcohol and smoking habits.

Diagnostic tests include electrocardiogram, echocardiography, and dosage of blood chemistry parameters such as B-type natriuretic peptide (BNP) urea and electrolytes, creatinine, complete blood count, and liver and thyroid function [19]. Echocardiography is essential to an exhaustive assessment of cardiac function. Traditionally, CHF is divided into three classes by the echocardiography measurement of left ventricular ejection fraction (LVEF): (i.) heart failure with a reduced ejection fraction (LVEF ≤ 40%), (ii.) heart failure with a mildly reduced ejection fraction (LVEF between 41% and 49%), and (iii.) heart failure with a preserved ejection fraction (LVEF ≥ 50%), which account for substantial differences in therapeutical management. In addition to LVEF, the echocardiographic assessment provides other significant parameters (ventricular volumes, systolic and diastolic functions, hypertrophy, etc.) that are essential for the correct phenotyping and following management [20].

#### 2.1.3. How Is It Related to Other Cardiovascular Diseases?

CHF is a complex and heterogeneous syndrome frequently associated with other cardiovascular diseases like coronary artery disease (CAD), the leading cause of CHF. CAD symptoms often overlap with those of heart failure; therefore, the onset of new signs and symptoms like dyspnea and fatigue may also indicate ischemic myocardial dysfunction [21].

Rhythm conduction disturbances, like atrial fibrillation and CHF, frequently coexist due to common pathophysiological mechanisms such as structural heart abnormalities, volume, pressure overload, and alterations in neurohormonal system activation [22].

Arterial hypertension can also be a significant risk factor for CHF if not adequately treated. Elevated blood pressure evokes an adverse remodeling of ventricular chambers and an augmentation of diastolic pressure that may lead to myocardial dysfunction [23].

Moreover, other comorbidities such as iron deficiency, anaemia, sleep disorders, chronic obstructive pulmonary disease, chronic kidney renal failure, thyroid disorders, and liver failure can be clinically manifest with the same symptoms and signs of CHF without its instrumental criteria. However, these conditions may coexist with CHF, exacerbating and influencing the disease’s onset and course [24,25].

#### 2.1.4. Technology in Chronic Heart Failure and Cardiovascular Medicine

Advances in various technology sectors have turned technological innovations into trustworthy solutions providing reliable personalized care to augment burdened healthcare systems [7,8]. These devices can be used to measure vital parameters like electrical heart activity, blood pressure (BP), respiration rate, and oxygen saturation $\left(SpO_{2}\right)$, which are useful to determine the comorbidities that may indicate the onset or progression of CHF [26].

**Figure 1:**
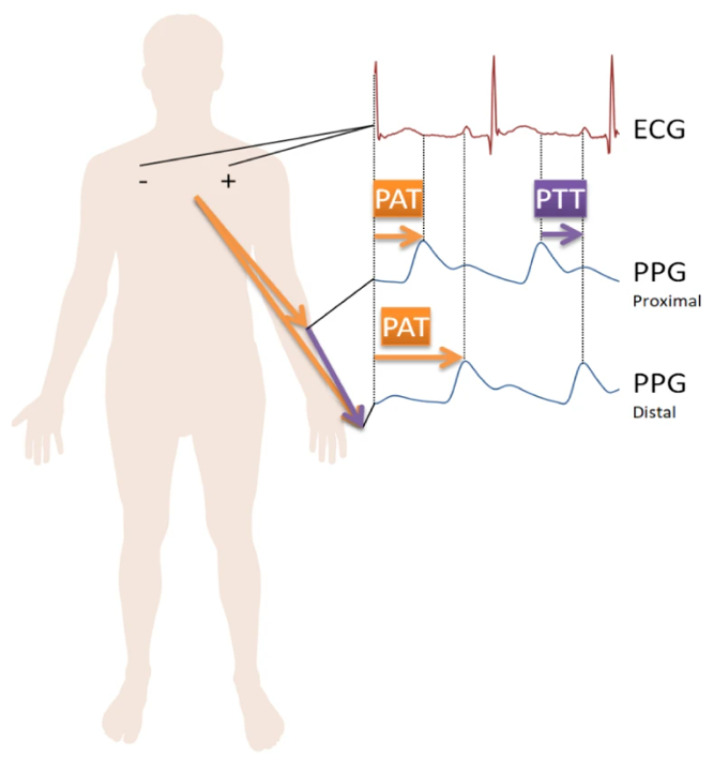
ECG and PPG signals and their corresponding on-body measurement sites [34].

Wearable, portable, or implantable devices can be adopted to measure the parameters of interest; however, owing to their unobtrusive and comfortable nature, wearable devices are the most popular contribution to the definition of the Internet of Medical Things (IoMT) [26,27]. Some of the most common wearable devices used in heart monitoring applications include sensors that can obtain electrocardiogram (ECG) and photoplethysmograph (PPG) signals [10,28,29,30] like those illustrated in Figure 1. ECG signals provide a cardiac pattern based on changes in the electrical potential generated by the heart [31], while PPG signals are provided by optical sensors that detect blood volumetric changes in peripheral arteries [32]. Additionally, due to advances in the sensing technologies used in wearable devices, clinically acceptable levels of accuracy can now be achieved, making medical practitioners more inclined to adopt technology solutions that aid patient care and management [33]. As mentioned earlier, in Section 1, AI algorithms allow for the accurate and intuitive analysis of health data to facilitate efficient at-home healthcare [3,5].

AI and ML technologies also improve patient care and the overall efficiency of cardiovascular medicine. According to several sources, AI and ML contribute to reducing the burden on medical practitioners through algorithms that perform various tasks like [10,11,28,35]:

- Identifying risk conditions by predicting health trends and acute events, thus enabling early warning and the early administration of treatment solutions;
- Providing personalized risk stratification, targeted therapies, and treatment solutions;
- Analysing chronic disease trajectories and response to administered therapies to provide recommendations for therapy adjustments;
- Optimizing hospital administration and scheduling systems;
- Optimizing surgical procedures;
- Optimizing pharmacological interventions;
- Improving doctor and patient communication.

## 3. Related Works

Due to the increased adoption of digital technologies in healthcare, various works have been published highlighting applied technologies; their impact on disease diagnosis, management, and treatment; and legislation implemented to regulate their use in clinical practices. Several studies on the diverse aspects of digital healthcare in cardiovascular medicine have been conducted to provide a picture of the current state of research, identify gaps, and future directions and potential solutions.

For instance, Tarakji et al. [10] provide an overview of legislative requirements and the state of commercial wearable technologies in cardiovascular medicine in the United States. They grouped the wearable devices into four categories, i.e., devices used to promote cardiac wellness and healthy living, pre-diagnostic and diagnostic devices, disease management devices, and devices developed for treatment in a survey about digital technology in clinical practices. The same study also provides an overview of AI and machine learning (ML) in cardiovascular medicine from a clinical perspective. The study focuses on techniques primarily applied to ECG signals for various applications, such as detecting paroxysmal atrial fibrillation (AFib) and low ejection fraction. The authors also indicate concerns regarding false positive or negative diagnoses that may cause stress and do more harm than good. They conclude that disease diagnosis and treatment algorithms must undergo rigorous clinical validation across multiple relevant demographic groups before adopting them to ensure trust. The work described here focuses on consumer wearables developed by large tech conglomerates and does not consider contributions to the field from academia.

Miller in [11] reviewed existing ML-enabled technology applications in cardiology to explain the fundamentals of AI and data science to medical professionals. The review discussed the various forms of ML and AI algorithms in CV medicine. The work covers AI and ML used to diagnose acute coronary syndrome (ACS) or perform ACS risk classification using various combinations of clinical data, lab results, and sensor data. The same study also discusses using ML, AI, and deep learning (DL) algorithms to predict mortality or adverse cardiac events based on imaging procedures such as contrast non-coronary computed tomography (CT) angiography or CT coronary calcium scoring or detect CAD using myocardial perfusion imaging (MPI). The survey focuses on presenting the concept of digital technologies for arrhythmia patients. However, it presents large-scale AI algorithms unsuitable for constrained wearable devices.

Potential heart conditions are predominantly detected using ECG signals, which reliably provide helpful information on heart function and heart health [10,28]. As a result, ECG measurement and interpretation techniques are the focus of many studies related to heart failure. For instance, the review by Chen et al. [36] outlines the various deep neural networks (DNNs) employed to perform ECG detection and classification. The study discusses DNNs like convolutional neural networks (CNNs) to process ECG signals for cardiovascular disease or acute cognitive stress detection. DNNs were also used to perform ECG signal compression and noise suppression to improve inference accuracy. The use of DL in wearable devices is briefly mentioned; however, the authors only nominate its application in wearable devices as a focus area for future studies. Hoffman et al. [28] also presented a survey of ML techniques applied to ECG signals to diagnose heart conditions. The 2020 survey by Hoffman et al. [28] discussed ML solutions to detect cardiac arrhythmias; identify coronary artery diseases (CAD); or unmask long QT syndrome, a disorder related to the heart’s electrical activity. The same study also reviews the use of ML algorithms to extract ECG signal features to diagnose heart failure, identify heart failure conditions, and infer developing heart conditions and pathologies. In the works they analyzed, ECG signals collected from wearable devices are transferred elsewhere for processing. Therefore, they identified the need for ML algorithms deployed in wearable devices to process ECG signals and improve the accuracy of the results they provide. In addition to ECG, several other wearable sensors can be used to determine the clinical signs and symptoms of CHF. As a result, a survey limited to AI applied to ECG analysis does not adequately provide the state of research and innovations related to edge AI in wearable devices used in the CHF domain.

As mentioned earlier, in Section 1, concerns about security and privacy cause clinicians and patients to hesitantly adopt medical technology. They are unsure how their data are used or who can access it. This concern promoted the adoption of digital ledger technologies (DLTs) such as blockchain to provide the required reassurance providing transparent and reliable data storage and exchange rules and infrastructure [37,38]. A survey by Xie et al. [39] gives a broad overview of how blockchain, wearable devices, and AI technologies can be combined to provide chronic-disease-management infrastructures. The survey focuses on the contribution of AI and big data to providing early diagnosis and prevention of chronic diseases using data collected by wearable and portable devices. However, it does not tackle the implementation of AI algorithms in wearable devices.

The studies summarized in this section reveal that the contributions of edge AI solutions in wearable architectures for CHF management and prevention are not adequately presented and highlighted. Therefore, this work collates and evaluates published research demonstrating architectures that leverage wearable devices and edge AI solutions for CHF management and diagnosis. The works selected for this discussion principally focus on the AI and ML applied on constrained devices at the extreme edge, i.e., the wearable devices themselves or smaller devices nearby. This study also aims to identify technology evolutions and provide a reference point for future developments, and it suggests potential research directions from a clinical and technology perspective.

## 4. Research Methodology and Contributions

The method adopted in this study is a “systematic literature review” [40,41,42], which entails using a well-defined protocol to conduct a rigorous literature search. The following subsections describe our procedure to perform a comprehensive and significant study.

### 4.1. Research Questions

We first defined some research questions and expected goals as listed below to guide the search criteria and delineate the direction of the review. The questions are designed to guide the progression of the research. They facilitate extracting the most relevant information to highlight research gaps. The questions also provide insights into the critical components of the selected research field. This is the most crucial step of a systematic literature review.

RQ1: What role does edge AI play in wearable healthcare architectures for CHF management?
  This question aims to determine the purpose for which edge AI is implemented in wearables used in CHF-prevention and -management architectures.
RQ2: How is edge intelligence applied in wearable-based intelligent healthcare architectures supporting CHF diagnosis, prevention, and management?
  This question provides an overview of existing edge AI methods and techniques that have been thus far adopted for CHF management, diagnosis, and prevention frameworks.
RQ3: How can edge AI contribute to developing interactive patient-centric solutions?
  This question demonstrates how users interact with intelligent systems containing wearables and edge AI technologies and the user services they offer.
RQ4: How do wearables and edge AI technologies affect the role of medical practitioners in chronic heart failure patient treatment and management?
  This question highlights the clinical significance of wearable and edge AI technologies in technologies applied to the CHF context. This question also aims to determine if existing works provide solutions to empirically quantify the contribution of the implemented edge intelligence solutions to the overall CHF-prevention, -management, or -diagnosis technology solutions. The goal is to provide insight into how the effect of these innovative technologies on patients and medical practitioners is evaluated and quantified.

### 4.2. Information Sources

To obtain the scientific sources used to perform this study, we selected multidisciplinary and domain-specific scholarly and scientific databases in line with the context of our research. We selected Scopus and Web of Science (WOS), the largest multidisciplinary scientific databases [43], as the first two reference databases. Considering the nature of this study covering the technical and medical domains, we also selected two databases, PubMed and IEEE Xplore, containing peer-reviewed publications from the domains. PubMed is the leading database for peer-reviewed medical and biological sciences publications [44], and IEEE Xplore is the most cited engineering and computer science academic database [45] to which we have access. By selecting these specific databases, we obtain peer-reviewed scientific articles tackling the subject from a multidisciplinary perspective, including the technical and clinical points of view. The database selection was also motivated by the global coverage they each provide. Then, based on the scope of the study and research questions specified in the preceding sub-section, we defined a set of keywords with which we defined a search query.

### 4.3. Inclusion and Exclusion Criteria

This study focuses on wearable sensors used in conjunction with edge AI in technology architectures applied to a chronic heart failure use case. Therefore, the principal elements of the study are:

1. Wearable sensing devices;
2. Edge AI;
3. Chronic heart failure.

We, therefore, defined a query (Listing 1) that includes synonyms of the main keywords to guarantee an exhaustive search intercepting all the articles relevant to our study.

**Listing 1.** Search Query.

(Edge OR on-device OR distributed OR embedded OR constrained OR tiny OR
FOG OR Mist) AND (``artificial intelligence’’ OR intelligence OR {AI} OR
``machine learning’’ OR {ML} OR ``deep learning’’) )AND (``wear *’’ OR
 worn) AND( (``cardi*’’ OR ``heart*’’ OR ``heart failure’’ OR ``chronic
heart failure’’) OR (``hospit*’’))


**Figure 2:**
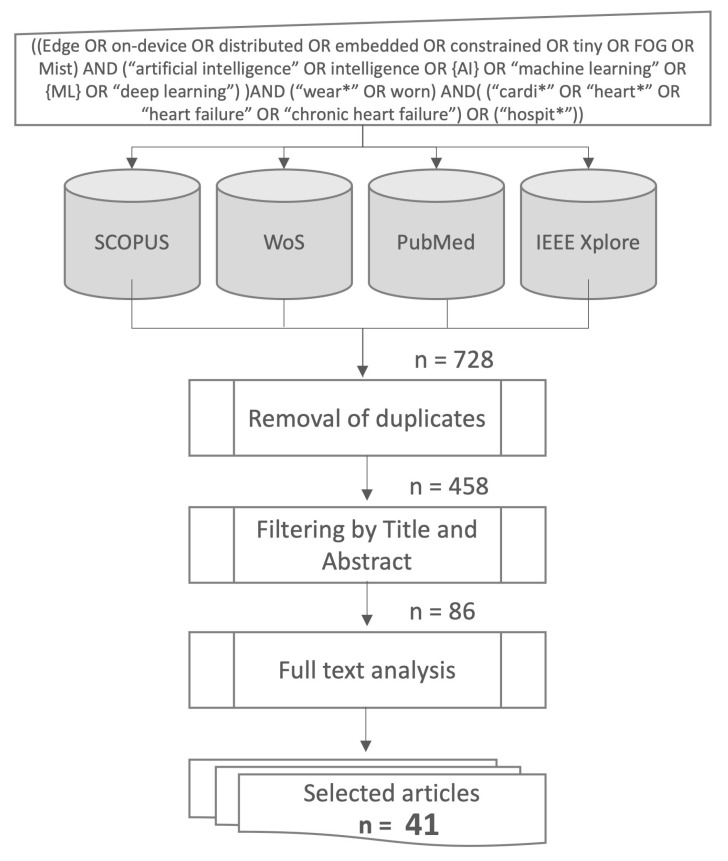
Flow diagram: methodology summary.

As summarized in Figure 2, following the search of all databases performed on 10 November 2022 using the defined query, a total of 728 articles published in English were obtained (Table 1). Many duplicates existed in this first tally because some scientific articles were indexed in multiple databases. Therefore, several duplicate removal iterations were performed.

In total, 565 articles were retained for further analysis after the first duplicate elimination iteration. Due to minor punctuation differences in paper titles in different databases, some duplicates were not identified using the duplicate removal method used in the previous step. Another elimination method that considers punctuation differences was implemented, and 107 duplicate articles were discarded, leaving a total of 458 articles. The typical systematic review screening procedure first employs title and abstract screening to identify relevant sources [42,46]; therefore, after removing all duplicates, the article titles and abstracts were used to select the relevant articles. During the first title and abstract elimination round, 372 additional papers irrelevant to our study were eliminated. Finally, 86 articles were selected for full-text analysis, and from that analysis, 41 articles were identified as relevant to our research and therefore selected for this study. Our study is limited to articles published on or before the 10 November 2022.

## 5. Results

This section highlights the main concepts related to the edge AI-powered wearable technologies used for CHF prevention, diagnosis, and management as presented in the articles selected for this study. The section provides a comprehensive discussion of articles obtained from the literature search described in Section 4 in an attempt to answer the questions outlined in Section 4.1. The first part of the discussion focuses on the application scenarios and technology-specific details presented in the analyzed articles in response to RQ1 and RQ2. The second part of this section focuses on the clinical and human elements of the solutions in response to RQ3 and RQ4. RQ3 aims to provide insight into user interaction with the systems and how edge AI affects this interaction. RQ4 highlights the methods used to quantify the effect of wearable devices with edge AI on the role of medical practitioners in CHF management, diagnosis, and prevention.

### 5.1. Application Scenarios

Many publications emphasize the importance of wearable technologies to continuously monitor and manage patients diagnosed with cardiovascular diseases like CHF or to facilitate early detection and treatment. The literature we analyzed reflects that arrhythmia detection using ECG signals is the main focus of most wearable technologies implementing edge AI, while PPG signals are the second most popular sensor for arrhythmia detection in CHF-prevention frameworks. In some of the architectures presented, multiple types of arrhythmia are automatically identified and classified based on cardiac patterns known to represent their occurence. However, some applications are designed to perform the more basic binary task of detecting the presence or absence of specific types of arrhythmia.

It is also estimated that about 50% of all deaths attributed to CHF are classified as sudden deaths, most of which are caused by ventricular arrhythmias [4]. Therefore, arrhythmia-detection solutions are crucial to CHF management architectures. Additionally, arrhythmias are common in CHF patients leading to disease progression and patient deterioration [4]. As a result, the early detection and monitoring of arrhythmias can allow for the timely administration of life-saving treatment.

One of the earlier arrhythmia-detection frameworks [2], along with the work described in [47], detected the presence or absence of atrial fibrillation (AFib), one of the most common cardiac arrhythmias [47,48,49]. The authors of [50] also defined a wearable system for MI early detection and prediction that confirms the presence or absence of potential MI-signaling heartbeats. Several other works also provide results that perform anomaly detection or only indicate the presence or absence of abnormal heart rhythms [6,30,51,52,53,54]. Amirhahi and Hashemi [55] also define a binary classifier to differentiate ventricular ectopic beats (VEBs) from non-VEBs.

Several arrhythmia detection applications, however, classify ECG signals into five classes, i.e., regular non-ectopic beats, supraventricular ectopic beats, VEBs, fusion beats, and unknown beats [3,5,56,57,58,59]. In Scire et al., however [60], all the above-mentioned beats except unknown beats are considered for classification. Another 5-class beat classification identifies normal (N), R-on-T premature ventricular contraction (Ron-T PVC), premature ventricular contraction (PVC), supraventricular premature or ectopic beat (SP or EB), and unclassified beat (UB) [61,62]. Other works determine the varying number of arrhythmia that can be used to detect heart malfunction and provide specific intervention insight. For instance, Meng et al. [63] identify premature ventricular contractions (PVCs) and supraventricular premature beats (SPBs). On the other hand, Abubakar et al. [64] use the classification of 13 different types of arrhythmias to determine abnormal cardiac rhythms and evaluate cardiac health. The work defined in [65] presents a classifier that identifies 17 classes of arrhythmias, while [49,66] distinguish atrial fibrillation (AFib) from normal rhythms, noise, and other rhythms. The work described in [67] also defines an algorithm trained to distinguish 26 different types of heart rhythms, including AFib, atrial flutter (AFlutter), premature atria contraction (PAC), or paroxysmal supraventricular tachycardia (PSVT), that can be used to define specific intervention options.

Patients with reduced LVEF are sometimes equipped with wearable cardioverter defibrillators (WCDs) to detect arrhythmic events and provide an appropriate high-energy shock to restore normal sinus rhythm [68]. WCDs are potentially harmful if not properly tuned. Therefore, Mazumder et al [68] defined an algorithm to classify shockable and non-shockable rhythms to contribute to a computer-aided shock-optimization model for preventing fatal ventricular arrhythmic propagation.

Huang et al. [69] instead took a different approach, implementing a framework to improve the care of patients implanted with bioresorbable vascular scaffolds (BVS) stents. Their framework uses wearable devices to track patients’ vital parameters, including BP, HR, and SpO2, to keep the doctor apprised of the patient’s condition to enable timely recommendations for follow-ups or corrective surgery if needed. In this framework, the wearable device is not equipped with edge AI; however, imaging devices equipped with edge AI are used in stent positioning and tracking algorithms when a follow-up is ordered.

Aside from monitoring arrhythmias, edge AI has also been applied to wearable-based BP measurements [70]. In other cases, edge AI is applied to preprocess or clean ECG or PPG signals used as classification algorithm inputs [29,55,60]. A summary of the application scenarios discussed in this section is provided in Table 2.

### 5.2. Deploying AI in Wearables

The computational devices used in wearable electronics that measure useful biosignals and complementary signals have limited power, computing, and memory resources. Therefore, in most cases, the first step towards implementing AI algorithms on wearable devices or proximity-portable smart devices is designing large-scale end-to-end algorithms. The large-scale DL algorithms must then be transformed using model compression strategies to reduce the model size and obtain resource efficiency. In such cases, only offline inference is performed in wearable devices. Pruning, quantization, clustering, knowledge distillation, and low-rank approximation are typically used to compress models for constrained device implementation [53]. The following section discusses the model optimization methods used in the analyzed literature.

#### 5.2.1. Model Compression and Transformation

**Figure 3:**
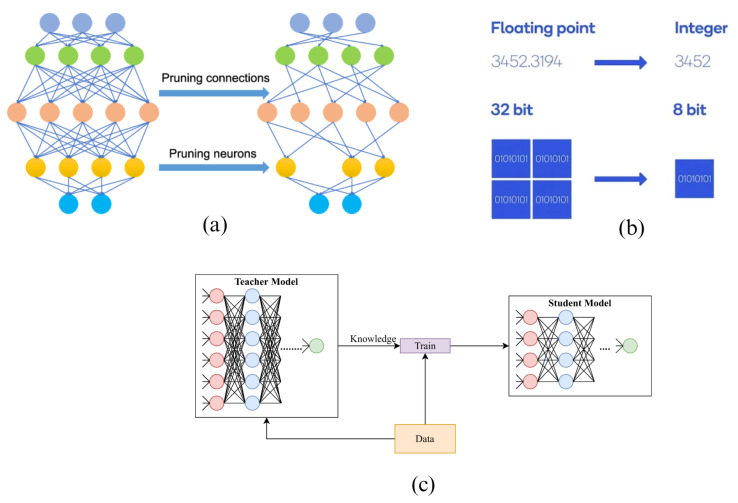
Model compression methods: (**a**) pruning, (**b**) quantization, and (**c**) knowledge distillation [75,76,77].

Some edge AI solutions in the selected literature repurpose large neural network (NN) architectures designed for unconstrained computational devices [52,53,61]. Model compression techniques like pruning, quantization, and knowledge distillation (Figure 3) can reduce large model resource requirements, thus optimizing them for constrained devices. Compressing a model by means of pruning involves deleting selected network parameters to achieve a reduced model without significantly affecting performance [53,71]. Quantization, instead, involves providing more compact representations of NN weights and/or activations to achieve [67]. Finally, knowledge distillation allows for the transfer of knowledge, such as weights and biases, from a larger model to a smaller one lacking the resources to learn them, allowing it to mimic the behavior of the model with higher complexity [49]. Large-scale models are either compressed or transformed in the analyzed works using automated frameworks like TensorFlow Lite (TFLite) [72]. Examples of this automatic model compression method are described in [52,53]. These works perform ECG anomaly detection in constrained devices using automatically compressed algorithms. ResNet and Mobilenet algorithms were utilized in [53], while a fully connected Deep Autoencoder was adopted in [52]. The model compression in [53] reduced the storage requirement by 99.9 % from 743 MB to 76 kb at the cost of a mere 1.2% accuracy reduction from 98.4% to 97.2%. The model presented in Ingolfsson et al. [61] instead compared several deployment frameworks, including TFLite [72], DORY [73], and [74] to compress a temporal convolutional network (TCN) to facilitate on-device inference. Accuracy losses of 0.2% and 0.4% were observed.

Other works define specialized model compression techniques to allow the implementation of NN in wearable devices. For instance, the authors of [58] explain a novel multi-stage pruning technique that reduces model complexity and improves runtime while preserving performance. The described method is tested against benchmarks and outperforms all other evaluated pruning mechanisms. In [3], a quantized CNN deployed on a Cortex M4 CPU is defined to provide arrhythmia classification. In this work, medical professionals can select the data processing mode from three distinct options. The first mode allows the device to transmit the measured ECG directly to the cloud storage. In mode 2, the wearable device only performs R peak detection; thus, the doctor is notified only when the heart rate exceeds a preset threshold. The final mode allows for the wearable device to classify the ECG signal into one of five classes defined by the training algorithm. The dynamic runtime configuration modes allow doctors to select the most helpful information based on patient needs. Instead, the work described in [66] employs 8-bit fixed point quantization to allow ECG classification in a constrained device using an LSTM RNN. The same authors slightly modified the network to improve compression and performance [49]. They used knowledge distribution and symmetric fixed-point quantization to reduce the model’s size. After knowledge distillation and quantization, a newer model, 43% smaller than the previous model, is obtained.

In contrast to the algorithms developed for ECG signals, the work defined in [30] defined an 8-bit quantized DNN that uses PPG signals to detect arrhythmia. In this work, the root mean square (RMS), skewness (SK), and kurtosis (KU) features are selected as inputs to the DNN that differentiates normal beats from abnormal beats.

The authors of [65] combine the quantization and algorithm-design approaches to define an arrhythmia classification wearable edge AI algorithm. The work defines a 1D-CNN architecture that is compressed using pruning and an adaptive loss-aware quantization (ALQ) method for deployment in wearable devices based on an application-specific integrated circuit (ASIC). The ALQ determines the quantization resolution of each CNN layer based on its sensitivity to limit the loss of accuracy due to compression. The ALQ method aimed to reduce model size while avoiding performance degradation; therefore, the result was a hardware-friendly algorithm with better accuracy and compression rate than other compression models.

Beyond identifying the presence or absence of abnormal heart rhythms, Ran et al. [67] define a supervised deep convolutional neural network (DCNN) to automatically classify 26 different heart rhythm classes. The authors implement pruning and integer quantization algorithm-hardware co-optimization techniques to allow inference on a constrained field-programmable gate array FPGA device that can be embedded in a wearable device. The hardware and algorithm co-optimization allows for accelerated inference and real-time functionality while maintaining performance. The tested algorithm uses 10s of ECG data and requires 2.895s inference time. The implemented integer quantization allows for low-bit integer operations, significantly reducing memory requirements, power consumption, and inference time. The pruned and quantized algorithm satisfactorily classifies ECG signals with an average F1 score of 0.913, superseding the 0.831 F1 score obtained by ECG physicians with more than 12 years of experience. Table 3 provides a summary of the compression models discussed in this section. The table also provides the processors onto which the models are deployed.

#### 5.2.2. Signal Conversion, Algorithm Design, and Modification

In addition to the quantization and pruning techniques, other methods used to improve resource efficiency and optimize AI models for constrained wearable devices include signal conversion [6,51,64], algorithm design and modification [48,59], and decision-based configuration [50]. Table 4 provides a tabulated summary of the additional methods used to improve resource efficiency in the sources we analyzed.

Increasing the accuracy of complex-signal DL classification algorithms typically involves increasing the number of hidden layers in the NN architectures [59,78]. This consequently increases the model storage and computational requirements and the training data required to produce accurate results. To avoid this problem, the authors of [59] define a shallow, lightweight algorithm based on a CNN structure by incorporating domain knowledge into the algorithm design process. This method optimizes the first CNN layer based on ECG signal frequency properties. Additionally, the network performs calculations parallel to the CNN layers to extract ECG quantitative features based on clinical knowledge to improve the detection of underlying pathologies. The resulting model performed comparably to other CNN-based models but boasted a lower parameter count due to the model configuration. Another method used to reduce AI processing complexity is signal format conversion. For instance, [64] converted ECG sequences into binary images for a tenary neural network (TNN). The algorithm defined in [6] uses ECG signals converted into scalograms as input to the 2D-CNN. Although the AI algorithm is not implemented in the wearable device, it is only triggered when the wearable device detects an abnormal heart rate to save power. In [51], ECG signals are converted to binary images and used as input to a binarized anomaly detection 2D CNN. The binary image resolutions determine the performance and resource requirements; therefore, in this case, a low-power (LP) and a high-performing (HP) model are selected for implementation. The LP model displayed a 91.7% F1 score compared to the HP model’s 86.8%. The two models allow for dynamic power-F1-score trade-off.

AI configurations traditionally used exclusively for specific applications like the natural language processing (NLP) transformer architecture can be modified and adapted for biosignal analysis as depicted in [63]. A fussing transformer-based model is adapted for ECG signal analysis by eliminating the decoder and modifying the model’s input embedding and self-attention parts. The resulting model incorporates a CNN architecture at the input to enhance feature extraction and replaces the fussing transformer self-attention with a lightweight depth-wise convolution architecture to improve the algorithm memory efficiency and classification accuracy. The authors of [63] nominate the possibility of applying their algorithm on wearable devices; however, implementation details are not provided.

The final algorithm modification technique employed to allow edge AI on wearable devices is presented in [79]. This method involves a multilevel partitioned CNN algorithm distributed between the wearable device and a cloud server. The deeper levels are only triggered if the wearable component detects anomalous behavior. This method contributes to the energy efficiency of the wearable device by reducing the complexity of the on-device model and reducing the communication frequency between the device and the cloud component.

**Table 4:** Tabulated summary-signal conversion, algorithm design, and modification.

Ref.	Model	Modification
[59]	KecNet-CNN based model	Domain knowledge-optimized 1st CNN layer merged with parallel ECG quantitative features based on clinical knowledge
[64]	Tenary neural network (TNN)	ECG sequences converted to binary images
[6]	2D-CNN	ECG signals converted to scalograms
[51]	2D-CNN	ECG sequences converted to binary images
[63]	Fussing transformer	Decoder eliminated-modified input embedding (CNN architecture) and replaced self-attention with a depth-wise convolution
[79]	CNN	Partitioned layers-First 2 layers in the wearable device-Last 3 layers in cloud

#### 5.2.3. Deployed As-Is

Other AI algorithms are naturally suited for constrained device implementation due to their native resource efficiency configurations. The algorithms deployed in their original form presented in the literature analyzed in this work are summarized in Table 5. Saadatnejad et al., for instance, [48], defined an RNN-based algorithm that can be directly implemented on portable/wearable devices without compression to classify several arrhythmias. The algorithm is tested on three devices: (i) the Moto360 AndroidWear smartwatch, (ii) NanoPi Neo Plus2, and (iii) Raspberry Pi Zero. This algorithm is trained by combining local and global datasets to improve personalization. However, like most other implementations, the wearable can only perform inference. Besides the traditional second-generation DNNs like CNNs or RNNs, third-generation spike neural networks (SNNs) are also well suited for low-power implementation, as demonstrated in [55].

Whereas preprocessing techniques like discrete wavelet transform (DWT) or similar R peak detection algorithms [6,50,64,80] are typically applied to the ECG signal before NN classification, the SNN defined in [55] is designed to perform both pattern-recognition and -classification tasks. Amirshahi and Hameshai optimized the SNN learning rules to best suit ECG signals; therefore, the resulting SNN-based ECG beat classification is characterized by accuracy values comparable to second-generation DNNs and significantly greater power efficiency. Similarly, Scire et al. [60] evaluated a k-nearest neighbor KNN-based classifier to perform heartbeat or R peak detection and an LSTM NN to classify arrhythmias.

The authors of [47] additionally defined a resource-efficient ML-based AFib detection edge inference pipeline. In their work, a bonsai algorithm is used to perform the real-time detection of the presence or absence of AFib. Using a tree-based algorithm also allowed the authors of [50] to adopt a hierarchical classification algorithm to reduce the power consumption and timing requirements in wearable devices. Using all available features to make confident classification decisions is not always necessary. Therefore, the lower classification levels use fewer features to achieve confident predictions and maintain low computational expenses. Furthermore, the more computationally expensive higher levels requiring more features are triggered only when the required confidence level is not reached. This approach reduces the overall computational complexity of the model and therefore prolongs the battery life of the wearable device, thus improving the overall device performance. In this instance, the hierarchical classification with four classification levels enhances battery life to 155 h, 2.6 times higher than the 59 h obtained using the full classifier.

In contrast to most of the work discussed in this section, the authors of [70] defined an ANN to provide systolic BP (SBP), diastolic BP (DBP), and mean arterial BP (MAP) estimation from PPG arterial pressure waveforms. Unfortunately, initial calibration with a cuff-based BP measuring device is required; however, the ANN allows continuous personalized BP estimation because the algorithms are tailored for each individual.

#### 5.2.4. Neural Architecture Search

Other research focused on developing neural architecture search (NAS) methods to obtain DNN architectures suitable for processing biosignals in wearable devices. One example is the work in [81], where a NAS tool performs a computation cost and performance trade-off analysis to select the best quantized TCN configuration for PPG heart rate analysis. Similarly, the work in [82] defined a genetic-based algorithm that varies key NN parameters to search for low-overhead hardware-aware architectures from a DNN architecture space. The authors used ResNet-based architectures; therefore, the key parameters, in this case, were the number of ResNet blocks, filters, and LSTM cells. In their work, user requirements like desired quality performance and the expected number of arrhythmia classes, in addition to hardware constraints including memory and supported operations, are used to construct datasets and generate the NN architecture space, respectively. The selected low-overheard architectures can be further compressed using pruning and quantization techniques. The NAS method defined in [82] enables the development of DNNs customized for anomaly detection or monitoring the recurrence of specific underlying conditions.

#### 5.2.5. Automated AI deployment

As mentioned earlier in this section, model-compression and device-optimization techniques can be performed through open-source tools and frameworks. These tools facilitate the automated generation of hardware-specific optimizations for efficient on-device inference. Some tools adopted in the works analyzed here are presented in Table 6. The table results show that automatic model compression tools and frameworks are not widespread in CHF-prevention and -management solutions, leveraging wearables that implement Edge DL algorithms.

### 5.3. Datasets

From the articles, we analyzed in this review, the MIT-BIH [83] arrhythmia dataset is the most popular choice for training the ECG arrhythmia classification AI algorithms, as displayed in Table 7. However, several open-source and proprietary databases have also been used to provide algorithm training and verification data.

The following table summarizes the databases used in the works analyzed in this study. The table shows that the implementation of edge AI is inherently skewed towards ECG analysis, as depicted by the recurrence of the MIT-BIH in most entries. As mentioned in Section 1, ECG analysis only provides a partial picture of patient health. From this observation, we infer that the lack of datasets containing multiple relevant wearable-based biosignals limits the diversity and, consequently, the capabilities of applied wearable edge AI technology. For this reason, we posit that creating adequately populated datasets containing all measurable CHF-relevant data is paramount to developing more well-rounded solutions. These datasets would include multiple biosignals related to CHF that can be measured by wearable devices and used to train models to facilitate the creation of interactive and complete patient-centric solutions. Other databases containing complex physiological signals that could be used to develop clinically acceptable edge NN architectures based on wearable devices for CHF-prevention and -management solutions can be found on Physionet [84].

Creating datasets on ill patients is a time-consuming endeavour, as described in [67], and is plagued by ethical issues; therefore, collaborations between medical professionals trained in patient care and interaction and technology experts are essential to the success of innovation in critical and chronic disease management.

**Table 7:** Tabulated summary-datasets.

Ref	Datasets
[64]	MIT-BIH Creighton University database. Reducing false alarms in ICU-PhysioNet/Computing in Cardiology Challenge 2015 dataset-G. Clifford et al.
[2]	MIT -BIH Atrial fibrillation database + Machine Learning Repository at University of California
[5]	MIT-BIH
[6]	MIT-BIH
[3]	MIT-BIH Arrhythmia dataset
[51]	MIT-BIH
[57]	MIT-BIH Arrhythmia database
[65]	MIT-BIH
[30]	PPG type 4
[49]	CinC2017–2017 Computation in Cardiology Challenge
[82]	MIT-BIH ECG dataset CU ventricular arrhythmia data set
[53]	Korea University Anam Hospital in Seoul, Korea,
[68]	Creighton University ventricular tachycardia database (CUDB) MIT-BIH Malignant ventricular arrhythmia database (VFDB)
[66]	2017 Computation in Cardiology Challenge
[55]	MIT-BIH ECG arrhythmia database
[61]	ECG5000
[47]	MIT-BIH Atrial Fibrillation DataBase Computing in Cardiology Challenge 2017 Database Ventricular fibrillation database
[70]	MIMIC-III Waveform Database from PhysioNet
[63]	Personalized database, CPSC2020, and MIT-BIH
[60]	MIT-BIH dataset
[52]	ECG5000
[62]	“BIDMC Congestive Heart Failure Database”
[85]	Unspecified ppg database
[80]	MIT-BIH
[67]	Privately collected dataset (training) Public China Physiological Signal Challenge (CPSC 2018) dataset (verification)
[59]	MIT-BIH arrhythmia (MITD-AR) QT database (QTDB)
[48]	MIT-BIH
[58]	MIT-BIH arrhythmia
[50]	Physiobank-PTB diagnostic ECG database
[56]	MIT-BIH arrhythmia (MITD-AR) QT database (QTDB)

### 5.4. Interactive Services

Efficient user experience is crucial to the success and acceptance of any activity of daily life or wearable technology. As such, communication with stakeholders is paramount to assessing efficiency and impact. However, most of the works discussed here focus on developing devices and algorithms and do not mention the service delivery and interaction with patients or medical practitioners.

The work presented in [15] provides insight into the requirements for person-centric heart monitoring systems obtained following consultations with cardiologists and cardiac specialists. The primary concerns mentioned in the article emphasized the need for development frameworks to be characterized by:

- Devices that are easy to use.
- Instruments that facilitate efficient communication between doctor and patient, i.e., easy-to-read data visualization instruments.
- Frameworks with combined biosensors and patient medical history.

In [64], system interaction is limited to physician alerts generated when an anomaly is detected. Similarly, Tiwari et al. [5] presented a system that allowed physicians to visualize the measured data and output classifications through a smartphone app. Finally, the work presented in [3] enabled physicians to configure the type of data they want to receive and the notification receipt frequency.

Interactions between patients and physicians are barely mentioned in the wearable-based architectures discussed in the study. Investigating extensive future developments focusing on the impact of combining the power of wearables and AI on patient–physician interactions would significantly contribute to obtaining a more substantial answer to RQ3 and RQ4.

## 6. Discussion and Research Opportunities

This section summarizes the primary outcomes and lessons learned from this review by highlighting the central answers to the established research questions. In addition, it sheds some light on remaining open issues and discusses the potential research opportunities emerging from the review.

The burden placed on healthcare infrastructures due to the cyclic and frequent hospitalization of CHF patients has increased significantly over the last few years [4,25]. With the evolution of medicine from reactive healthcare to predictive, preventive, personalized, and participatory (P4) medicine [86], the focus has shifted towards incorporating technology to provide patient-specific tailored solutions and therapies. A vital asset towards this endeavour is adopting wearable devices to continuously monitor patent physiological signals to help provide medical practitioners with a large number of data with which they can make informed decisions.

Section 5.1 details the contributions of wearable devices equipped with AI to CHF patient care, as presented in the analyzed literature, and summarizes application scenarios in response to RQ1. The more prevalent application scenarios involve detecting abnormal heart functions, such as ventricular arrhythmias, common among CHF patients [4]. Detecting arrhythmias or abnormal heart rhythms provides early warning to prevent and, in some instances, slow down disease progression. However, the potential contributions of AI in wearables equipped with ECG, PPG, or other sensors to evaluate different parameters like LVEF that are widely used in the clinical evaluation of CHF patients still need to be investigated.

Obtaining a complete assessment of CHF patients that compares to clinical practices requires monitoring multiple parameters, including respiration and oxygen saturation, which provide doctors with information about fatigue that is an important factor for determining patients’ New York Heart Association (NHYA) class. The NYHA class allows doctors to determine the severity of CHF in an individual as well as the individual’s risk of death [4]. Adopting architectures with sensors that can provide the information required and allow for the automatic evaluation of the patient NYHA class would significantly improve the efficiency of wearable devices applied to CHF management. As observed in the analyzed results, most of the presented edge AI solutions use ECG or PPG sensors to provide signals for arrhythmia or anomaly detection. Therefore, adopting other sensing technologies that provide contextual heart health information like e-tattoos and smart patches like those presented in [87,88,89] in wearable CHF patient monitoring architectures could contribute to developing robust wearables acceptable to clinicians.

Based on the observations made in Section 5.3, only a small subset of the datasets containing physiological signals from CHF have been used to date. New solutions could therefore use more complex datasets like those available on Physionet [84] to provide complete patient digital twins. In contrast to hospital or lab ECG evaluation, wearable devices collect ECG recordings in uncontrolled conditions, causing signal artefacts. Therefore, further studies are also needed to evaluate, optimize, and standardise methods used to handle artefacts and improve the classification of signals acquired by wearable devices [29,47].

The discussion in Section 5.2 also effectively responds to RQ2. In a nutshell, several approaches have been taken to allow for the implementation of AI algorithms in wearable devices applied to CHF-prevention and -management infrastructures. The end-to-end pipeline of AI deployment currently involves two steps, i.e., algorithm design and training, followed by model optimization to enable offline inference on constrained devices. In some cases, as shown in Table 3, compression techniques are automatically applied to computationally expensive algorithms using open-source tools like TFLite and GapFlow. However, in most instances, model optimization is performed using techniques tailored for the required outputs and selected application data. Many advances have been made in improving AI implementation on constrained devices. Furthermore, most works we studied use DNNs in their architectures because they allow for automated feature extraction. DNN automatic feature extraction eliminates the need for domain knowledge when developing algorithms. However, in the medical field, explaining the decision-making steps taken by an algorithm is essential in medical applications due to regulations and ethics [90]. It is essential to note that the AI paradigm boasts an eclectic selection of algorithms and techniques that can be adapted for the healthcare domain, i.e., the analysis of biosignals and disease markers using domain expertise. Therefore, assessing the adoption of explainable AI or ML techniques that rely on manual feature extraction could be an effective research direction. As a result, more collaborations among technology experts and clinicians would provide high-performance and robust AI-powered wearable solutions to monitor CHF patients. Indeed, it is believed that patients actively involved in decision-making and therapy-management processes are more likely to respond better to therapies than passive patients [91]. Therefore, another potential future development in which new AI techniques can be used to promote patient involvement in the domain is implementing AI techniques (e.g., based on decision tree algorithms) that explain the predictions or the decisions made by the algorithm. In this way, patients can be involved in decision making and potentially provide feedback to improve the predictions. Developing architecture similar to the cognitive companion described in [92], with added edge AI-based, privacy, and efficiency preserving audio-visual patient interaction capabilities, would demonstrate a significant step forward in the future of trusted and patient-centred technology-based medicine.

Although the application of technology to prevent CHF and manage diagnosed patients is being tackled from many fronts, practical demonstrators and real-life testing remains challenging. In most cases, the development of edge AI in wearable devices is only mentioned as a future development, creating a significant divide between conceptualization and implementation. Therefore, a potential research direction would be performing dedicated pilot studies including patients, clinicians, and technology experts to propose solutions acceptable to all stakeholders.

Another important lesson from this study regards collaboration among technology innovators and medical professionals. Most of the works we analyzed only provide the technological point of view. As a result, most of these discussed solutions are not adopted by clinicians. Collaboration among technology experts and clinicians can facilitate research that produces fascinating outcomes and practical solutions that both communities could appreciate.

However, the absence of collaboration is also evident among stakeholders of different domains and among experts in the same field. We noticed a divide among the works focused on the hardware and the software components. The software experts, in fact, mainly concentrate on developing dedicated frameworks with complete pipelines and interconnection through dedicated APIs. In contrast, hardware experts usually focus on hardware optimization, performance evaluation, etc. This division, unfortunately, sometimes leads to incomplete products and influences the users’ acceptance of innovative solutions. Therefore, future research directions should account for collaboration, not only among stakeholders operating in different domains but also among experts of the same area operating on different aspects (e.g., hardware and software).

Finally, based on our observations, we believe a comprehensive solution that leverages edge intelligence, IoT wearable sensors and user interfaces to provide a digital twin with two intercommunicating platforms could be an efficient CHF management solution. The two components of the digital twin would include (i.) a patient-centred digital twin based on virtual assistance solutions to support the patient and receive helpful feedback to improve predictions and treatment plans, and (ii.) a physician-centred digital twin solution to support medical professionals in their daily activities and help them to enhance the patients’ overall well-being, thus improving the efficacy of care and patient management. Such a solution must integrate edge AI technologies in wearables to provide distributed data elaboration and reduce the IoT data storage dilemma. However, cloud computing is still essential to developing complete infrastructures due to available technologies and scalability requirements.

## 7. Conclusions

Various innovative technologies have been proposed and applied to provide technology solutions contributing to CHF diagnosis, management, and prediction, reducing mortality and improving patient care. However, most available solutions leverage the cloud computing paradigm that is unfortunately affected by communication latency, privacy issues, etc. Despite the existence of several distributed computing scenarios, cloud computing still has an integral role in many application solutions. However, as IoT devices increasingly produce a large number of data, the need for applications implementing computing solutions based on reliable distributed intelligence hastens. We hypothesize that implementing AI in wearable devices can significantly contribute to the widespread use of decentralized computing. Fortunately, the adoption of wearable-based edge intelligence for CHF diagnosis management and prediction has been the subject of many research endeavours, as demonstrated in this study. However, to the best of our knowledge, the literature lacks a comprehensive analysis evaluating research about wearable solutions implementing edge AI to prevent and manage CHF.

Therefore, this study presented a systematic literature review discussing 41 articles related to edge AI techniques applied to wearable-based chronic heart-failure-prevention and -management infrastructures. A staggering 80% of the articles were published within the last two years, demonstrating growing interest in the subject.

Approximately 46% of the total number of articles analyzed in this survey describe the use of varied optimization techniques to enable the implementation of AI algorithms on resource-constrained wearable devices. In total, 68% of those articles highlight the application of model compression techniques on large-scale algorithms. The remaining 32% describe algorithms modified for constrained devices through signal conversion or applying domain knowledge-based modifications to the algorithms. Finally, only about 14% of the analyzed articles presented algorithms deployed without changes. Due to these observations, it can be concluded that efficient optimization techniques are essential for deploying AI algorithms in wearable devices. Only four articles we analyzed adopted tools to perform hardware-specific optimizations on predefined models automatically. Therefore, further investigation is required to determine why. The MIT-BIH dataset is used in over 50% of the articles that provided information about the datasets used. Therefore, also, in this case, further investigation is required to determine the reason for this considering the availability of datasets containing heart failure patient physiological parameters.

Several existing open issues, as discussed in Section 6, exist in this field; therefore, there is still much work to be done to define clinically acceptable technology solutions based on wearable devices for diagnosing, monitoring, and managing CHF patients.

In conclusion, artificial intelligence is only at the beginning of its application in the medical field, and its potential is barely perceived. The strength of wearable technologies in medicine lies in their low impact on the patient’s daily routine, which significantly improves healthcare. Equipping patients with intelligent wearable technologies also gives them a central role to play in their clinical assessment and makes them active in the therapeutic process, thus improving their chances of positive outcomes.

## Figures and Tables

**Table 1:** Search results.

Database	No. of Articles
Scopus	273
WOS	118
PubMed	155
IEEEXplore	182

**Table 2:** Tabulated summary-application scenarios implementing edge AI in wearable devices.

Ref.	Application Scenario	Output/Classification
[64]	Abnormal cardiac rhythms	Thirteen arrhythmia classes
[3,5,56,57,58]	Arrhythmia classification	Five classes-normal non-ectopic beats, supraventricular ectopic beats, VEBs, fusion beats, and unknown beats
[60]	Arrhythmia classification	Four classes-normal non-ectopic beats, supraventricular ectopic beats, VEBs, and fusion beats
[61,62]	Arrhythmia classification	Five classes-normal (N), R-on-T premature ventricular contraction (Ron-T PVC), premature ventricular contraction (PVC), supraventricular premature or ectopic beat (SP or EB), and unclassified beat (UB)
[65]	Arrhythmia detection	Seventeen rhythm classes
[2,47]	Atrial fibrillation detection	Binary-presence or absence
[70]	BP estimation	BP values
[69]	BP, HR, and SpO2 tracking for BVS maintenance	Binary-normal or abnormal
[63]	Cardiac rhythm classification	Two classes-premature ventricular contractions (PVCs) and supraventricular premature beats (SPBs).
[6,30,51,52,53,54]	Cardiac rhythm anomaly detection	Binary-normal or abnormal
[49,66]	Cardiac rhythm classification	Four classes-atrial fibrillation (AFib), normal rhythms, noise, and other rhythms.
[67]	Heart rhythm classification	Twenty-six heart rhythm classification +
[50]	Myocardial Infarction	Binary-presence or absence
[68]	Shockable rhythm detection for WCD control	Two classes-shockable and non-shockable rhythms
[29,55,60]	Signal cleaning and preprocessing	-
[55]	Ventricular ectopic beats (VEBs) detection	Binary-true or false

**Table 3:** Tabulated summary-model compression and transformation.

Ref.	Model Type	Processor	Optimization
[64]	Tenary neural network	ASIC wearable embedded processor	Tenary quantization
[53]	ResNet and Mobilenet	-	TensorFlow Lite-auto pruning and quantization
[52]	Autoencoder	nrf52840-cortex M4 CPU	TensorFlow lite-auto compression and optimization
[61]	Temporal convolutional network (TCN)	GWT GAPuino-GAP8 RISC-V	GapFlow/TFLite compression and optimization
		B-L475EIOT01A STM32L4-Cortex M4 CPU	
[58]	CNN	-	Multistage pruning
[3]	1D CNN	ST Sensor tile-cortexM4 CPU	8-bit quantization
[66]	CNN-LSTM	nRF52832 -cortexM4 CPU	8 bit fixed-point quantization
[49]	CNN-LSTM	nRF52832-cortexM4 CPU	Knowledge distillation and symmetric fixed-point quantization
[30]	DNN	Arduino BLE Nano 33 Sense-Cortex M4	int8 quantization
[65]	1D CNN	ASIC	Pruning and adaptive loss-aware quantization (ALQ)
[67]	DCNN	Xilinx Zynq XC-7Z020 FPGA-(ARM Cortex-A9 + Artix-7 FPGA)	pruning and quantization

**Table 5:** Tabulated summary-deployed as-is.

Ref.	Model	Device/CPU
		Moto360 androidwear device
[48]	LSTM	NanoPi Neo Plus2
		RaspberryPi zero
[55]	Spike neural networks	ARM Cortex A53
[60]	Beat detection KNN and classification LSTM	-
[47]	Bonsai	Raspberry Pi 3 Model B-Cortex-A53
[50]	Random forest	Cortex M3
[70]	Artificial neural network (ANN)	EFM32 Leopard Gecko ARM Cortex-M3

**Table 6:** Automation tools and frameworks.

Ref.	Tool/Framework	Quantization Tool/Library	Device Specific Code Generator	Device	Processor
[61]	GapFlow	TFLite	AutoTiler	GWT GAPuino	GAP8 RISC-V
	NEMO/DORY	NEMO	DORY	GWT GAPuino	GAP8 RISC-V
	TF	TFLite	-	B-L475EIOT01A STM32L4	Cortex M4
	CUBE.AI	TFLite	CUBE.AI	B-L475EIOT01A STM32L4	Cortex M4
	CUBE.AI	CUBE.AI	CUBE.AI		
[52]	TFLite Micro	TFLite	-	nRF52840	Cortex M4
[62]	TF	TFLite	-		
[53]	TF	TFlite	-		

## Data Availability

Not applicable.

## Abbreviations

The following abbreviations are used in this manuscript:

AFib	Atrial fibrillation
AI	Artificial intelligence
ALQ	Adaptive loss-aware quantization
API	Application program interface
ASIC	Application-specific integrated circuit
BNP	B-type natriuretic peptide
BSV	Biore-sorbable vascular scaffolds
CAD	Coronary artery disease
CHF	Chronic heart failure
CNN	Convolutional neural network
CT	Computed tomography
CV	Cardiovascular
CVD	Cardiovascular disease
DL	Deep learning
DCNN	Deep convolutional neural network
DNN	Deep neural network
ECG	Electrocardiogram
EMR	Electronic medical records
LSTM	Long short-term memory
LVEF	Left ventricular ejection fraction
MI	Myocardial infarction
ML	Machine learning
MPI	Myocardial perfusion imaging
NN	Neural network
P4	Predictive, preventive, personalized, and participatory
PAC	Premature atria contraction
PPG	Photopletysmogram
PSVT	Paroxysmal supraventricular tachycardia
RMS	Root mean square
SoC	System on chip
TCN	Temporal convolutional network
TFLite	Tensor flow lite
TNN	Tenary neural network
